# Light and Electron Microscopy of the European Beaver (*Castor fiber*) Stomach Reveal Unique Morphological Features with Possible General Biological Significance

**DOI:** 10.1371/journal.pone.0094590

**Published:** 2014-04-11

**Authors:** Natalia Ziółkowska, Bogdan Lewczuk, Wojciech Petryński, Katarzyna Palkowska, Magdalena Prusik, Krystyna Targońska, Zygmunt Giżejewski, Barbara Przybylska-Gornowicz

**Affiliations:** Department of Histology and Embryology, Faculty of Veterinary Medicine, University of Warmia and Mazury, Olsztyn, Poland; 2Institute of Animal Reproduction and Food Research of Polish Academy of Sciences, Olsztyn, Poland; Federal University of Rio de Janeiro, Brazil

## Abstract

Anatomical, histological, and ultrastructural studies of the European beaver stomach revealed several unique morphological features. The prominent attribute of its gross morphology was the cardiogastric gland (CGG), located near the oesophageal entrance. Light microscopy showed that the CGG was formed by invaginations of the mucosa into the submucosa, which contained densely packed proper gastric glands comprised primarily of parietal and chief cells. Mucous neck cells represented <0.1% of cells in the CGG gastric glands and 22–32% of cells in the proper gastric glands of the mucosa lining the stomach lumen. These data suggest that chief cells in the CGG develop from undifferentiated cells that migrate through the gastric gland neck rather than from mucous neck cells. Classical chief cell formation (i.e., arising from mucous neck cells) occurred in the mucosa lining the stomach lumen, however. The muscularis around the CGG consisted primarily of skeletal muscle tissue. The cardiac region was rudimentary while the fundus/corpus and pyloric regions were equally developed. Another unusual feature of the beaver stomach was the presence of specific mucus with a thickness up to 950 µm (in frozen, unfixed sections) that coated the mucosa. Our observations suggest that the formation of this mucus is complex and includes the secretory granule accumulation in the cytoplasm of pit cells, the granule aggregation inside cells, and the incorporation of degenerating cells into the mucus.

## Introduction

There are two species of beavers: the European beaver (*Castor fiber*) and the Canadian beaver (*Castor canadensis*). Both species are herbivorous and belong to a small group of mammals with diet containing a large quantity of woody plants. Beavers are the second largest rodents after capybaras.

The gross morphology of the Canadian beaver stomach was first reported in 1868 [1]. Very few anatomical and histological descriptions of this organ have been published since then [2], [3], [4]. The stomach of the Canadian beaver is distinguished from that of most mammals by the presence of the cardiogastric gland (CGG), a gland-like structure located along the lesser curvature near the oesophageal entrance. To date, no publications have described the morphological features of the European beaver stomach.

Since the histology and ultrastructure of the stomach of both *Castoridae* family members are not well known, we sought to characterize this organ in the European beaver using light and electron microscopy. Gross morphological investigations were also performed. We identified several unusual and previously unknown features of the beaver stomach.

## Materials and Methods

### Ethics Statement

The study was carried out in a strict accordance with Polish laws of animal welfare. Beaver capture and euthanasia were approved by the Regional Directorate for Environmental Protection in Olsztyn, a government institution responsible for management of wildlife in the Warmia and Mazury Voivodship of Poland, where the study was performed. Animal captures were allowed due to beaver overpopulation in this area and were conducted by a specialized team from the Polish Hunting Association. The experimental protocol including animal euthanasia was approved by the Local Ethical Commission for Experiments on Animals at the University of Warmia nad Mazury in Olsztyn (Permit Numbers 22/2007, 33/2009) and the III Local Ethical Commission for Experiments on Animals at Warsaw University of Life Sciences (Permit Number 11/2010). All possible efforts were made to minimize animal suffering.

### Animals

The study was performed using ten male and ten female European beavers (*Castor fiber*) between two to six years of age. The animals were captured in their natural habitat in north-eastern Poland during the fall. Beavers were euthanized within three hours of capture by exsanguination under deep anesthesia. Anesthesia was induced by intramuscular administration of 60 mg of xylazine (Sedazin, Biowet Puławy, Poland) and 300 mg of ketamine (VetaKetam, VetAgro, Poland). The stomachs were removed immediately after the heart stopped and used for anatomical and histological studies (n = 12) as well as ultrastructural investigations (n = 8).

### Gross anatomical and histological studies

Each stomach was measured and cut along the greater curvature. The mucosa was then gently flushed with warm saline (30–35°C) and photographed using a Nikon D700 digital camera and an appropriate objective (Nikon, Japan).

Samples for histological analysis were obtained from: 1) the stomach wall of the lesser and greater curvatures, including the oesophageal entrance and the pyloric sphincter, at ca. 5 cm intervals; 2) the central areas of the cranial and caudal stomach walls from the fundus, corpus, pylorus, and the corpus-pylorus border; and 3) the CGG. The CGG was cut into parts and all of them were prepared for light microscopy. Tissues were fixed in 10% buffered formalin for 48 hours, washed, dehydrated, and then embedded in paraffin. Samples were cut into 4-µm thick sections by a HM 340E microtome (Microm, Germany) and stained with hematoxylin and eosin (H&E) [5]. Sections were observed and photographed using an AxioImager motorized light microscope and an AxioCam MRc5 digital camera (Carl Zeiss, Germany).

The cellular composition of the proper gastric glands was studied using the histological slides images obtained by a MiraxDesk scanner (Carl Zeiss, Germany) and AxioVision 4.8 software (Carl Zeiss, Germany). Cells were counted in 500000 µm^2^ areas containing longitudinal sections through the gastric glands. Five areas from each studied part of the stomach per animal were analyzed.

To measure mucus thickness, samples of the stomach corpus wall were taken from six animals (three males and three females), frozen in liquid nitrogen, sectioned at −25°C using a HM 560 Cryostat (Microm, Germany), and then stained with H&E [5]. Wet, freshly prepared slides were photographed using differential interference contrast, which satisfactorily visualized the mucus layer and pit cells. The measurements were carried out on four sections taken from different parts of each studied stomach.

The area of the cardiac region was estimated by measurements from histological sections (area width) and freshly dissected stomachs (internal stomach perimeter at the oesophageal entrance). The calculations were performed according to the following equation: (area width at the lesser curvature + area width at the greater curvature)/2× internal stomach perimeter at the oesophageal entrance. The areas of the fundus/corpus and pyloric regions were measured using photographs of the whole mucosa in AxioVision 4.8 software (Carl Zeiss, Germany). The location of the corpus-pylorus border was confirmed by histological studies.

### Ultrastructural studies

Mucosa samples from the fundus, corpus, and pylorus as well as mucosa and submucosa samples from the CGG were collected for electron microscopy within three minutes after heart stopping. Tissue pieces were fixed (2 hours, 4°C) in a mixture of 1% paraformaldehyde and 2.5% glutaraldehyde in 0.2 M phosphate buffer (pH 7.4). Samples were then washed, postfixed in 2% osmium tetroxide (two hours at room temperature), and embedded in Epon 812. Semi-thin sections were cut from each block of tissue, stained with toluidine blue, and examined to identify regions for electron microscopy. Ultrathin sections were contrasted with uranyl acetate and lead citrate and then examined using a Tecnai 12 Spirit G2 BioTwin transmission electron microscope (FEI, USA) equipped with two digital cameras: Veleta (Olympus, Japan) and Eage 4k (FEI, USA).

## Results

### Anatomy

**Figure 1:**
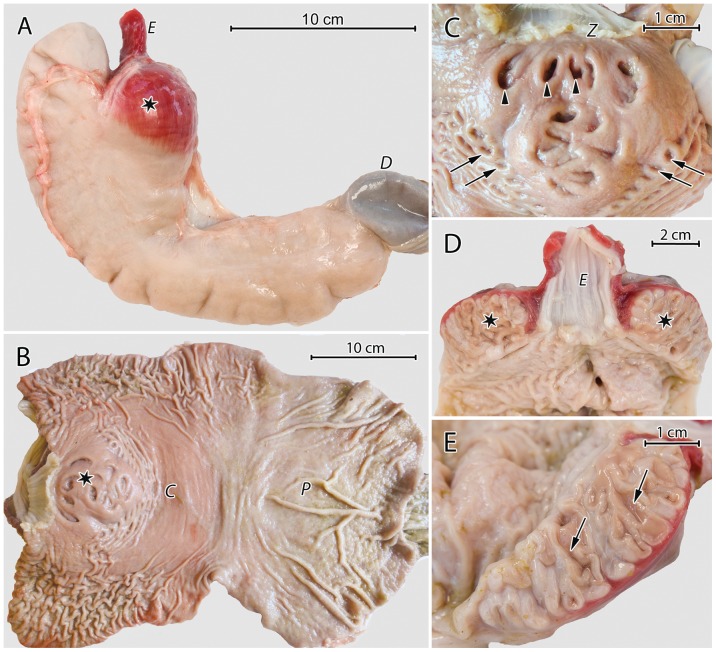
Gross morphological features of the European beaver stomach. (A) An external view of the stomach. Asterisk: the CGG on the lesser curvature; *E*: oesophagus; *D*: duodenum. (B) The mucosa after cutting the stomach along the greater curvature. Note differences in mucosal colour between the corpus - *C* and pylorus - *P*. Asterisk: the CGG. (C) The CGG viewed from the stomach lumen. Note the large crater-like (arrow heads) and small (arrows) orifices. *Z*: gastroesophageal junction (D) The stomach cut along the lesser curvature. Note the continuity between the muscular coat of the CGG and the muscularis of the esophagus. Asterisk: the CGG; *E*: oesophagus. (E) Section of the CGG showing its internal organization. Arrows: branched tubes that open on the luminal mucosal surface.

The stomach of the European beaver was a unilocular, C-shaped sac with a length (measured along the grater curvature) of 28 to 40 cm and a perimeter of 22 to 30 cm at the corpus and 15 to 18 cm at the pylorus (Figure 1A). The CGG was located immediately behind the oesophageal entrance along the lesser curvature of the stomach; it was visible as a dark red, globular structure with a diameter of approximately 5 cm (Figure 1A).

The stomach mucosa formed numerous folds and was greyish-pink at the fundus and corpus, and grey at the pylorus (Figure 1B). The gastroesophageal junction was prominently serrated (Figure 1B, C). The CGG was connected to the stomach cavity by 36 to 40 orifices on its luminal surface (Figure 1B, C). Twelve were crater-shaped, 5 to 7 mm large, and located at the CGG's center. The remaining orifices were smaller, 1 to 2 mm large, and surrounded the area containing the larger apertures (Figure 1C).

The internal organization of the CGG was visible after cutting the stomach along its lesser curvature (Figure 1D, E). The gland was comprised of densely packed, branched tubes that opened at the luminal surface of the mucosa. The muscular coat of the CGG was continuous with the muscularis of esophagus (Figure 1D).

### Histology of mucosa and submucosa

The entire gastric mucosa was lined with the surface epithelium comprised of a single layer of columnar cells. Differences in mucosa organization allow to distinguish three separate areas in the stomach: the cardiac region, the fundus/corpus region which contained the CGG, and the pyloric region. The cardiac, fundus/corpus, and pyloric regions occupied 0.4%, 50.3%, and 49.3% of the mucosal surface, respectively.

The submucosa was formed by loose connective tissue and contained numerous blood vessels, submucosal plexus neurons, and scattered bundles of smooth muscle cells. With the exception of the CGG, the structure of submucosa did not significantly differ between the investigated stomach regions.

#### Cardiac region

**Figure 2:**
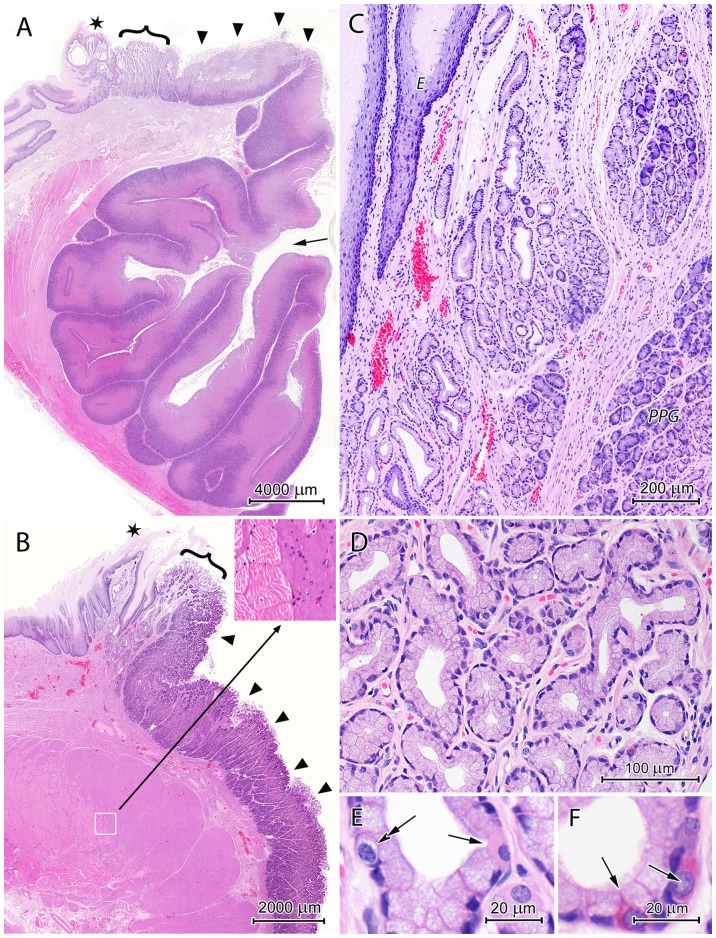
Histological structure of the cardiac region. (A) Longitudinal section through the lesser curvature near the oesophageal entrance. Note that the CGG is composed of branched invaginations of the mucosa into the submucosa. Arrow: one of CGG orifices; Asterisk: the gastroesophageal junction; Curly bracket: the cardiac region; Arrow heads: the corpus region. (B) Longitudinal section through the greater curvature near the oesophageal entrance. The insert shows the magnified border between the skeletal muscle fibers of the oesophagus and the smooth muscle cells of the stomach. Asterisk: the gastroesophageal junction; Curly bracket: the cardiac region; Arrow heads: the fundus region. (C) Section of the cardiac mucosa with clusters of cardiac glands separated by the connective tissue. The stratified squamous epithelium of the oesophagus (*E*) and the proper gastric glands - *PGG* are visible nearby. (D) Cardiac glands forming tubes with the irregular lumen and consisting primarily of mucous cells. (E) Mucous cells filled with secretory granules, parietal cell (arrow), and endocrine cell with pale cytoplasm (double arrow). (F) Endocrine cells with acidophilic cytoplasm (arrows) between mucous cells.

The cardiac region originated at the serrated gastroesophageal junction and formed an irregular mucosal ring with a width ranging from 0.9 to 2.3 mm (Figure 2A, B).

The gastric pits in this region were as deep as a third of the mucosa thickness. The surface epithelium was comprised of columnar cells with basally situated nuclei. The cytoplasm of these cells contained various numbers of pale secretory granules, depending on the localization of cells in the gastric pits. The apical surface of pit cells was covered by a thick layer of mucus (100–200 µm in paraffin sections).

The cardiac glands were loosely distributed throughout connective tissue of the lamina propria (Figure 2C). These glands were characterized by an irregular lumen and the presence of small and often cup-shaped branches (Figure 2C, D). The glands were comprised primarily of cuboidal mucous cells with flattened, basally-situated nuclei and foamy cytoplasm (Figure 2D, E, F). Stem cells with mitotic figures, endocrine cells with pale or acidophilic cytoplasm (Figure 2E, F), and individual parietal cells were also present (Figure 2E). The lamina propria contained a relatively dense network of blood vessels (Figure 2C, D).

The thick muscularis mucosae consisted of internal circular and external longitudinal layers of smooth muscle cells.

#### Fundus/corpus region

**Figure 3:**
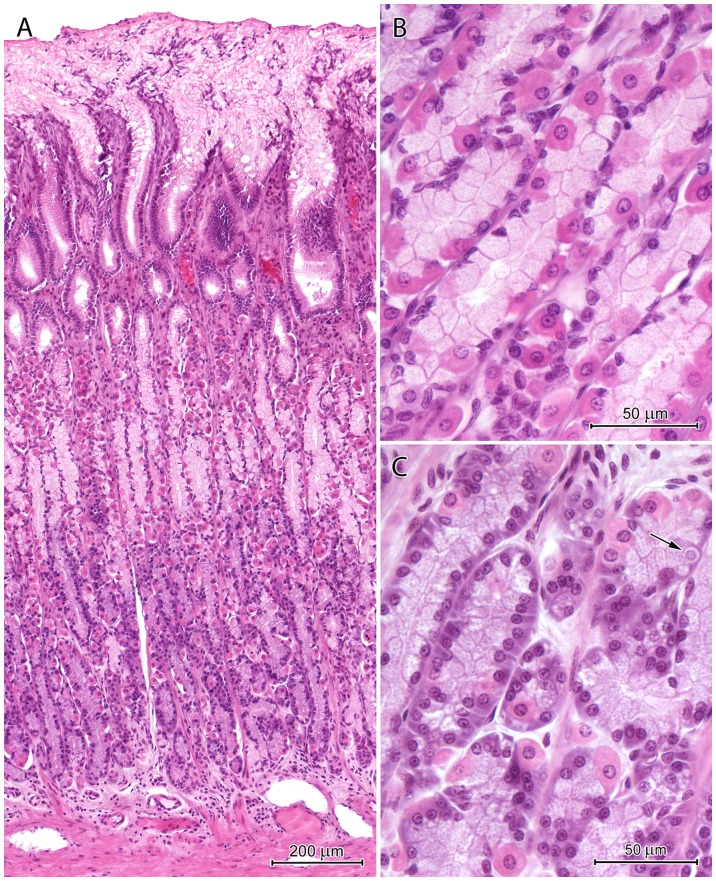
Histological structure of the corpus region. (A) Section through the mucosa of the corpus. Note the thick layer of the mucus that covers the mucosa and fills the gastric pits. (B) Necks of the proper gastric glands containing numerous, well-developed mucous neck cells and parietal cells. (C) Bases of the proper gastric glands consisting of numerous chief cells and parietal cells. Arrow: endocrine cell with pale cytoplasm.

**Figure 4:**
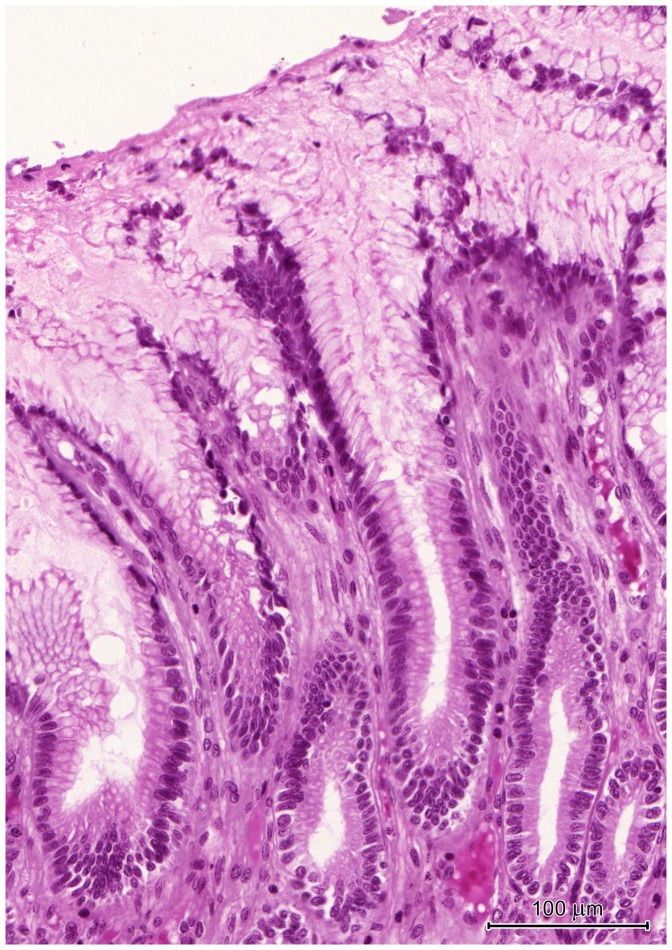
Surface epithelium cell morphology and mucus structure. Note differences in the appearance of cells that line various parts of the pits as well as the presence of cell debris in the mucus.

The mucosal surface in the fundus/corpus region was covered by a very thick layer of mucus that completely filled the upper parts of the gastric pits (Figures 3A, 4). The mucus reached a thickness of 950 µm in frozen, non-dehydrated sections and 400 µm in paraffin sections. The mucus was heterogeneous and contained cellular debris from exfoliated cells of the surface epithelium (Figure 4). Cellular debris was arranged in regular rows that ran towards the stomach lumen. Mucus was not visible at the bottom of the gastric pits in H&E-stained paraffin sections (Figures 3A, 4).

The gastric pits in the fundus/corpus region occupied approximately 20 to 25% of the mucosa thickness (Figure 3A). Morphology of surface epithelium cells varied by their localization in the gastric pit. Therefore, five zones could be distinguished in each pit (Figure 4). From the pit base, they were characterized by: 1) columnar cells with elongated nuclei and cytoplasm stained pink with eosin; 2) cells similar to those of the first zone but with weakly stained apical regions; 3) short, columnar cells with round, darkly stained nuclei and foamy cytoplasm; 4) cells with flat, darkly stained nuclei, foamy cytoplasm, and damaged apical surfaces; 5) cellular exfoliation resulting in contact between connective tissue and mucus.

**Figure 5:**
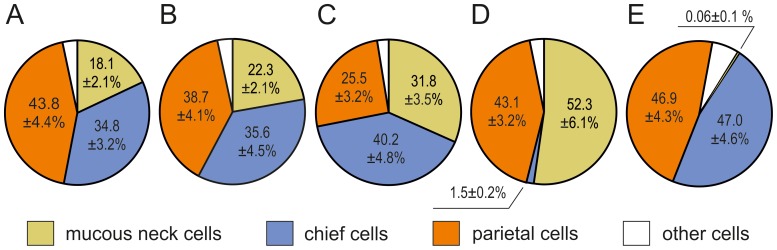
Cellular composition of the proper gastric glands. (A) Fundus mucosa. (B) Corpus mucosa at the greater curvature opposite to the GCC. (C) Mucosa surrounding the CGG orifices. (D) Corpus mucosa close to the pylorus. (E) The CGG. Cells were counted in ca. 500 000 µm^2^ areas that contained longitudinal sections through the gastric glands, as described in “Material and methods”. Total number of cells forming the glands was taken as 100%. The presented data are means and standard deviations (n = 12).

The lamina propria of the mucosa in the fundus/corpus region contained numerous proper gastric glands. These glands formed straight tubules that consisted of five cell types: stem cells, parietal cells, mucous neck cells, chief cells, and endocrine cells (Figure 3A). These cells were consistently distributed, and the isthmus, neck, and base of the glands could be easily distinguished (Figure 3A). Numerous mitotic figures were observed in stem cells of the isthmus. Parietal cells were present throughout the gland but were most abundant in the neck (Figure 3B, C). These cells were triangular or oval, approximately 18 to 26 µm, and had central nuclei and eosin-pink cytoplasm. Some parietal cells had two nuclei. Mucous neck cells were square or short columnar cells with height of 15 to 21 µm and width of 9 to 15 µm (Figure 3B). Typically, these cells were slightly thinner at the apex and broader at the base where the nucleus was located. The apical region of the mucous neck cells was filled with secretory granules, which were weakly stained by H&E. The basal region of the proper gastric glands was comprised primarily of chief cells (Figure 3B). These cells were short columnar (height: 14–20 µm, width: 7–10 µm) and dichromatic by H&E staining (Figure 3C). The lower, perinuclear regions of chief cells were darkly stained by hematoxylin, and the upper regions were filled with pale granules (Figure 3C). Endocrine cells were oval with weakly stained cytoplasm. There were no differences in cellular composition between proper gastric glands in the fundus (Figure 5A) and those in the middle part of the corpus (Figure 5B). Mucous neck cells were slightly more abundant in the mucosa surrounding CGG orifices (Figure 5C). The mucosa of the distal part of corpus, near the pylorus, contained glands that consisted primarily of mucous neck cells and parietal cells (Figure 5D).

Like that of the cardiac region, the muscularis mucosae of the fundus/corpus region was formed by two layers of myocytes.

#### CGG

**Figure 6:**
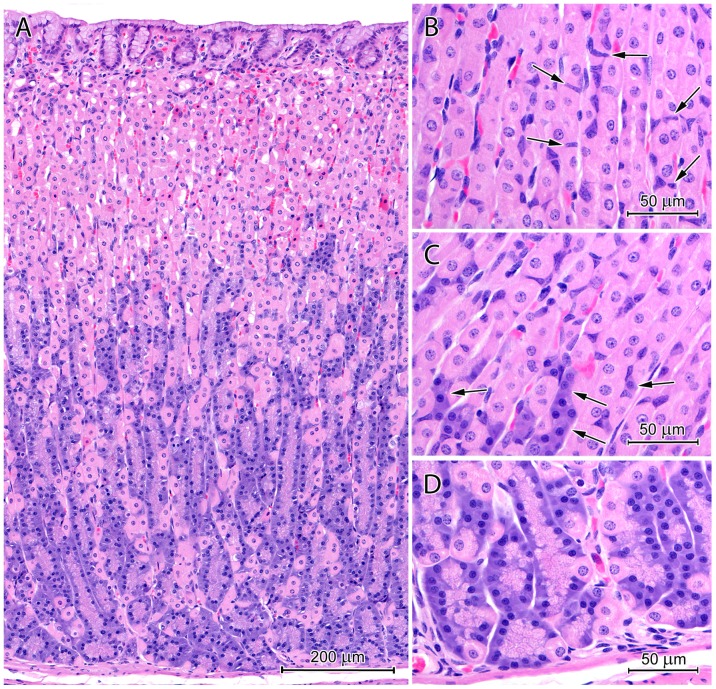
Histological structure of the CGG. (A) Mucosa forming tubular invaginations into the submucosa. Note the surface epithelium creating shallow foveolae and the proper gastric glands shaped like very long straight tubules. (B) The upper parts of the proper gastric glands formed primarily of parietal cells, which created rows with neighboring cells, and small undifferentiated cells (arrows). (C) Developing chief cells (arrows) at the border between the upper and lower parts of the glands. (D) The lower parts of the proper gastric gland formed primarily of chief cells. Large, triangular parietal cells are also visible. Note the thin layer of smooth muscle cells that comprise the lamina muscularis.

The CGG was formed by tubular, branched invaginations of the mucosa into the submucosa (Figure 2A). These structures were 1.2 to 2.0 cm in length and had diameters of 2.2 to 4.9 mm. The invaginations were tightly packed in the submucosa and reached the tunica muscularis. They were comprised of all three mucosa layers: the surface epithelium, the lamina propria with proper gastric glands, and the lamina muscularis (Figure 6A). The histology of the mucosa, which formed the invaginations was considerably different from that of the mucosa lining the gastric lumen in the corpus/fundus region and surrounding CGG orifices.

The surface epithelium of the CGG consisted of short columnar cells with basally-situated nuclei and weakly stained supranuclear material. It created shallow foveolae that were not deeper than 8% of the mucosa thickness and had a very narrow lumen (Figure 6A).

The proper gastric glands of the CGG were very long (0.8–1.3 mm), straight tubes (Figure 6A). The upper parts of these tubes were formed mainly by parietal cells, which created rows of neighboring cells (Figure 6B, C). Parietal cells were oval or pyramidal and ranged in size from 20 to 25 µm. Their cytoplasm was unevenly stained by H&E, which showed weakly stained cytoplasm parts surrounded by strongly acidophilic areas. Bi-nuclear cells were frequently observed in this cell population. Small, undifferentiated cells with oval, darkly stained nuclei and sparse cytoplasm were observed between the parietal cells (Figure 6B). Mucous neck cells were hardly observed and represented less than 0.1% of the cells forming the proper gastric glands in the CGG (Figure 5E). The basal parts of these glands consisted primarily of chief cells but also included many parietal cells (mostly triangular with a size of 25–30 µm) and a few endocrine cells, usually with pale cytoplasm (Figure 6D). Chief cell morphology was similar to that described above. Developing chief cells, with strongly basophilic cytoplasm were observed at the border between the upper and lower parts of proper gastric glands (Figure 6C). The number of chief and parietal cells in the proper gastric glands of the CGG was almost equal (Figure 5).

The muscularis mucosae, limiting from outside the invaginations, was created by a thin layer of longitudinally oriented smooth muscle cells (Figure 6D).

#### Pyloric region

**Figure 7:**
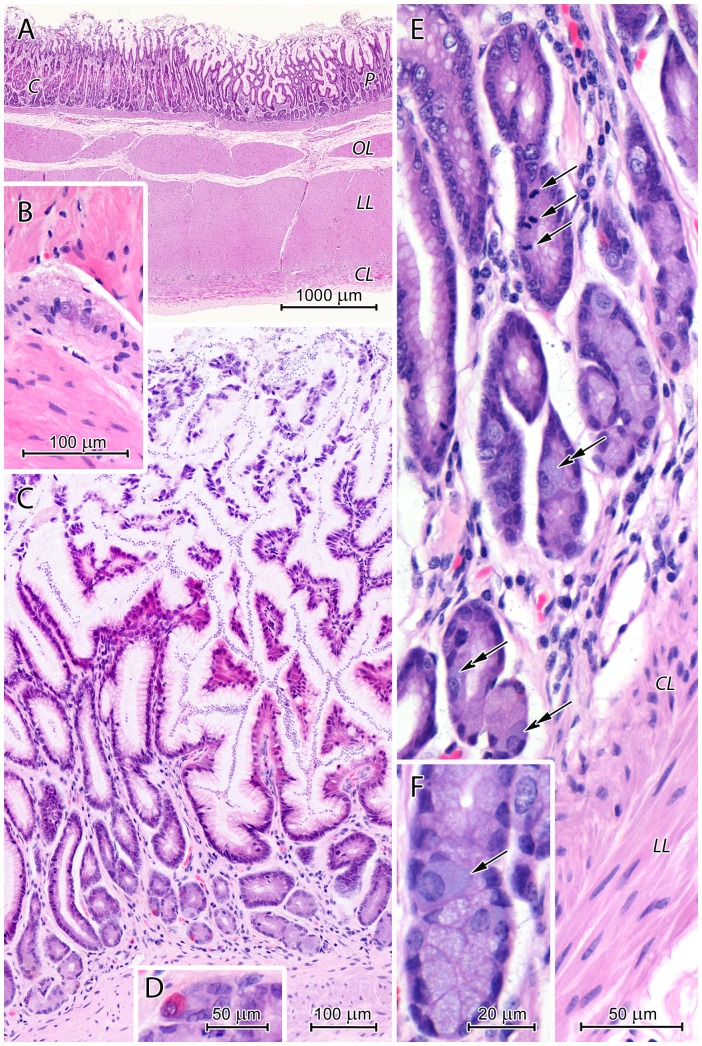
Histological structure of the pyloric region. (A) Section through the stomach wall at the border between the corpus - *C* and pylorus - *P*. Note the differences in shape and depth of gastric pits between the regions. The muscularis consisted of three layers: oblique - *OL*, longitudinal - *LL*, and circular - *CL*. (B) The myenteric plexus situated between the circular and longitudinal layers of the muscularis. (C) The pyloric mucosa with very deep, branched gastric pits and short pyloric glands. (D) Endocrine cell with strong acidophiclic cytoplasm in the pyloric gland. (E) The pyloric glands and the lamina muscularis of the mucosa consisting of circular - *CL* and longitudinal layers - *LL*. Note numerous mitotic figures (arrows) in the isthmus and the presence of cells with basophilic cytoplasm (double arrows). (F) Cell with basophilic cytoplasm (arrow) among mucous cells in the pyloric gland.

The border between the corpus and pylorus was sharp and clearly visible by both the gross morphological (Figure 1B) and histological studies (Figure 7A). The mucosa of the pyloric region formed strongly branched, very deep gastric pits, occupying about 80% of its thickness (Figure 7A, C). It was covered by a prominent layer of mucus between 30 and 150 µm thick in histological sections (Figure 7C). In contrast to the previously described region, the mucus did not completely fill the gastric pits. Surface epithelium structure was similar to that in the corpus, however in the pylorus the zone containing cells with foamy apical cytoplasm covered larger part of the gastric pits (Figure 7C).

The pyloric glands were very short and consisted of stem cells, mucous cells and endocrine cells (Figure 7C–F). Numerous mitotic figures were observed in stem cells forming the isthmus (Figure 7E). Mucous cells had round or flat nuclei and foamy, slightly basophilic cytoplasm (Figure 7F). Endocrine cells had strongly acidophilic or pale cytoplasm when stained by H&E (Figure 7D). Oval cells (10–12 µm×15–20 µm) with large, euchromatin-rich nuclei and homogeneous, basophilic cytoplasm were also observed (Figure 7E, F).

The muscularis mucosae was well-developed and formed by two layers of myocytes (Figure 7E).

### Histology of muscularis and serosa

**Figure 8:**
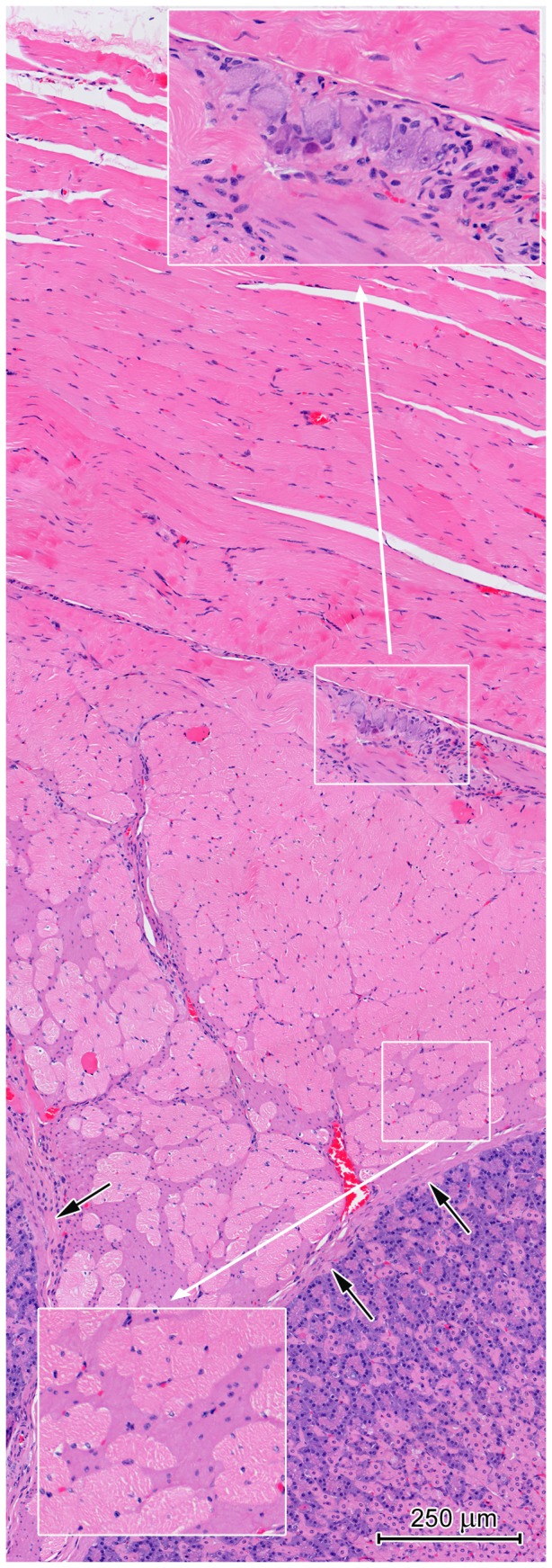
The muscularis covering the CGG. Note the external layer is comprised exclusively of skeletal muscle fibers while the internal layer consists of both muscle tissue types. The top insert shows the myenteric plexus. The lower insert shows the two types of muscular tissue in the internal layer. Arrows: the lamina muscularis.

The muscularis of the European beaver stomach was comprised of two types of muscle tissue: skeletal and smooth. Along the greater curvature, skeletal muscle tissue of the esophagus muscularis was replaced by smooth muscle at the gastroesophageal junction (Figure 2A). In contrast, along the lesser curvature, skeletal muscle tissue largely formed the muscularis that covered the CGG (Figures 2A, 8). The external longitudinal muscularis layer in this region of the stomach consisted exclusively of skeletal muscle fibers while the internal, circular layer was formed by both types of muscle tissue (Figure 8). In the remaining portions of the stomach wall, the muscularis was comprised of smooth muscle cells and had two (circular and longitudinal) or three (oblique, circular, and longitudinal) myocyte layers (Figure 7A). The two-layer muscularis was found in the cardiac region and at the end of the pylorus. The circular layer formed the pyloric sphincter. Numerous myenteric plexus neurons were located between the circular and longitudinal muscularis layers (Figures 7B, 8).

The exterior of the stomach was covered by a serosa consisted of simple squamous epithelium and loose connective tissue.

### Ultrastructure

#### Surface epithelium

**Figure 9:**
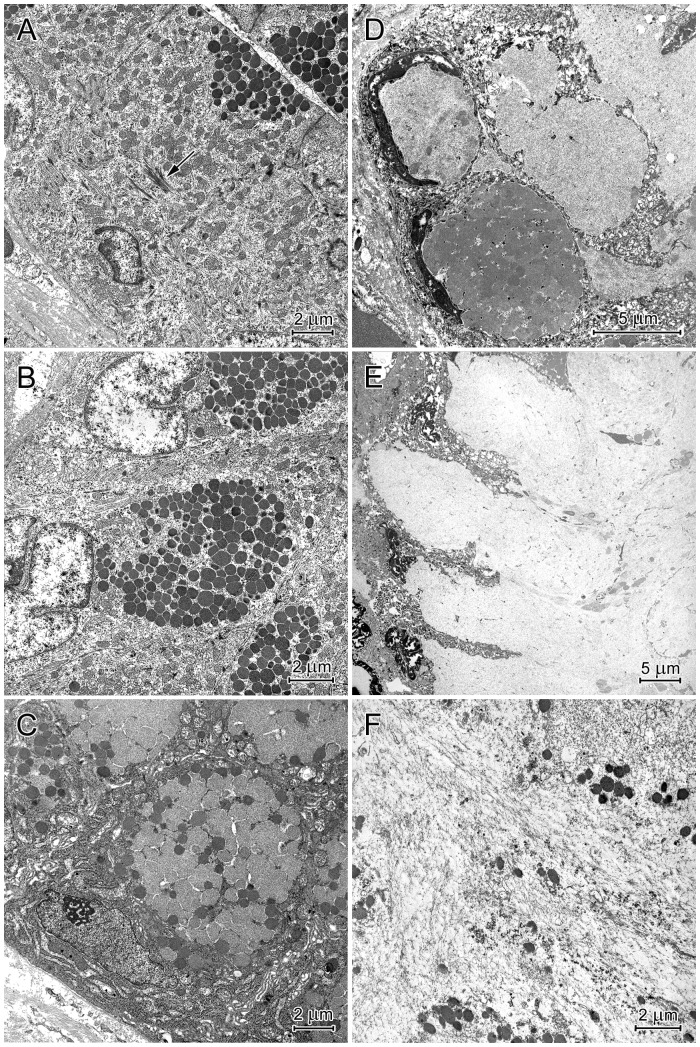
Ultrastructure of the surface epithelium cells in the basal (A), middle (B, C), and upper (D, E) regions of the corpus gastric pits and mucus structure (F). (A) Pit cells containing electron dense granules in their apical parts. Note the bundles of tonofilaments (arrow). (B) Cells with numerous electron-dense granules that form oval clusters in the supranuclear cytoplasm. (C) Cells with numerous granules that vary in electron density and aggregate with each other. (D) Degenerating cells with flattened nuclei and cytoplasm filled by the mucous mass. (E) Cells with destroyed apical regions. Note the mucus is partially released from the cell. (F) Mucus ultrastructure. Cell debris and fine fibrils are visible.

The structure of surface epithelium cells depended on their localization in the gastric pits. In the stomach corpus, the cells situated in the basal portion of the pit were columnar and had oval nuclei with deep nuclear envelope invaginations (Figure 9A). A small to moderate number of electron-dense granules were present in the most apical regions of the cells, and exocytosis of their content was frequently observed. These cells also contained numerous mitochondria, tonofilament bundles and formed finger-like connections with each other (Figure 9A).

The cells in the middle portion of the gastric pit were filled with numerous secretory granules that created a large, oval supranuclear cluster (Figure 9B, C). This accumulation was surrounded by a thin rim of cytoplasm, which separated the granules from the cell membrane. Granule morphology varied along the longitudinal axis of the gastric pit. Cells deeper in the pit had electron-dense, round or oval granules (Figure 9B). In addition to these granules, more superficial cells also contained large, irregularly shaped granules with moderate electron density, which resembled the “mucus droplets” of goblet cells (Figure 9C). As the number of granules with moderate electron density increased, the number of electron-dense granules decreased (Figure 9B, C). The large granules with moderate electron density merged to create a uniform mucous mass (Figure 9C). Parallel to the changes in granule morphology, cell nuclei became more and more flat.

Cells in the upper portion of the gastric pit had pyknotic nuclei and cytoplasm filled with a low to moderate electron-dense mucous mass (Figure 9D). In some cells, the borders between the granules forming the mucus mass remained visible. Cells at the top of the gastric pit had destroyed apical regions (Figure 9E). They were exfoliated and included in the mucus covering the stomach mucosa. The mucus contained cellular debris and fine fibrils (Figure 9F).

Similar pit cell morphology was also observed in the pylorus. Compared to the corpus, cells in the lower and middle regions of gastric pits in the pyloric region had more granules with variable electron densities. The granule content was released by exocytosis.

#### Proper gastric glands in the fundus and corpus mucosa

The isthmus of proper gastric glands in the fundus and corpus consisted of numerous stem cells and developing parietal cells.

**Figure 10:**
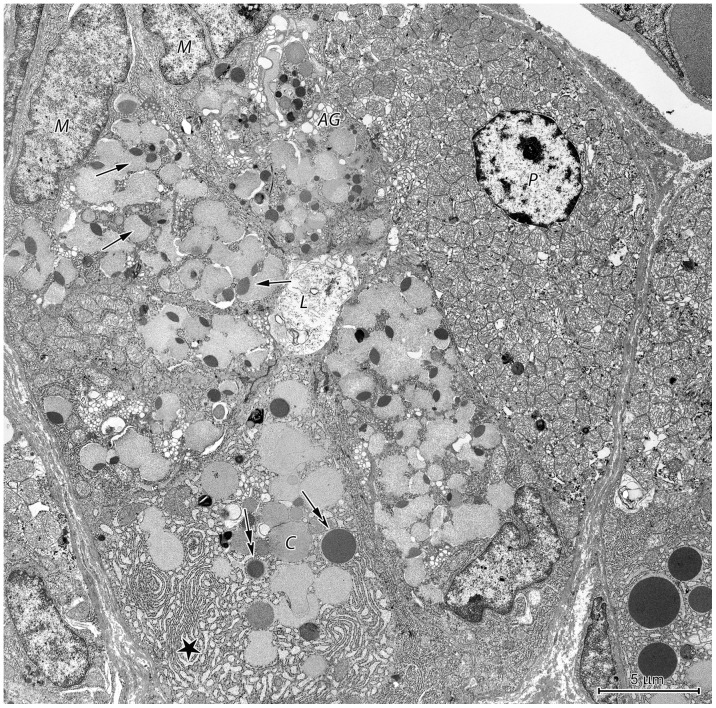
Ultrastructure of the neck of a proper gastric gland in the stomach corpus. Mucous neck cells - *M*, parietal cell - *P*, and differentiating chief cell - *C* surrounding the lumen of the gland - *L*. Note the bipartite secretory granules (arrows) and well-developed Golgi apparatus – *AG* present in the mucous neck cells as well as the numerous cisternae of rough endoplasmic reticulum (asterisk) and round, electron-dense secretory granules (double arrows) in the developing chief cell.

The neck was formed by mucous neck cells, parietal cells, and differentiating chief cells (Figure 10). Mucous neck cells were characterized by flat, euchromatin-rich, basally situated nuclei and numerous irregularly shaped, electron-lucent, supranuclear granules (Figure 10). A specific feature of these cells was the occurrence of granules comprised of both high and low electron density parts (i.e., bipartite). These granules were observed in all mucous neck cells that were examined. Mucous neck cells had a well-developed, supranuclear Golgi apparatus, mitochondria occurring alone or in clusters, and small amounts of rough endoplasmic reticulum (RER) cisternae, which were primarily distributed near the nucleus.

Differentiating chief cells could be distinguished from mucous neck cells by the presence of numerous long RER cisternae (Figure 10). In addition, these cells contained a few secretory granules with homogeneous electron-dense contents, which were distributed between electron-lucent granules (Figure 10). Parietal cells were characterized by numerous mitochondria and a well-developed tubulovesicular system (Figure 10).

**Figure 11:**
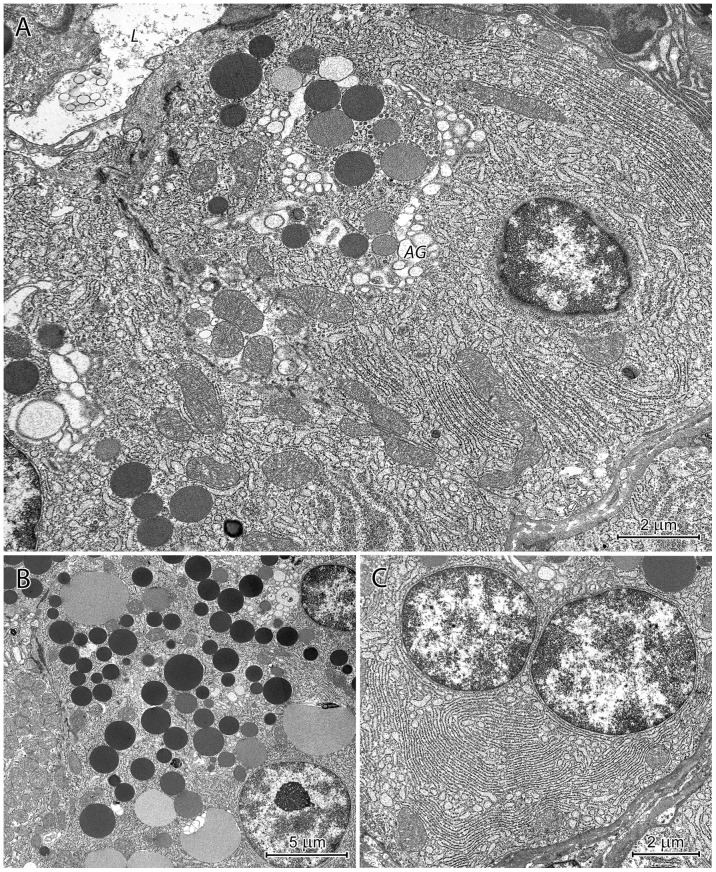
Ultrastructure of chief cells in the proper gastric gland of the corpus mucosa. (A) Bipolar organization of chief cells. Note the well-developed Golgi apparatus (*AG*). L: lumen of the gland. (B) Numerous secretory granules, varying in size and electron density, located in the supranuclear cytoplasm. (C) Well-developed rough endoplasmic reticulum in the basal region.

**Figure 12:**
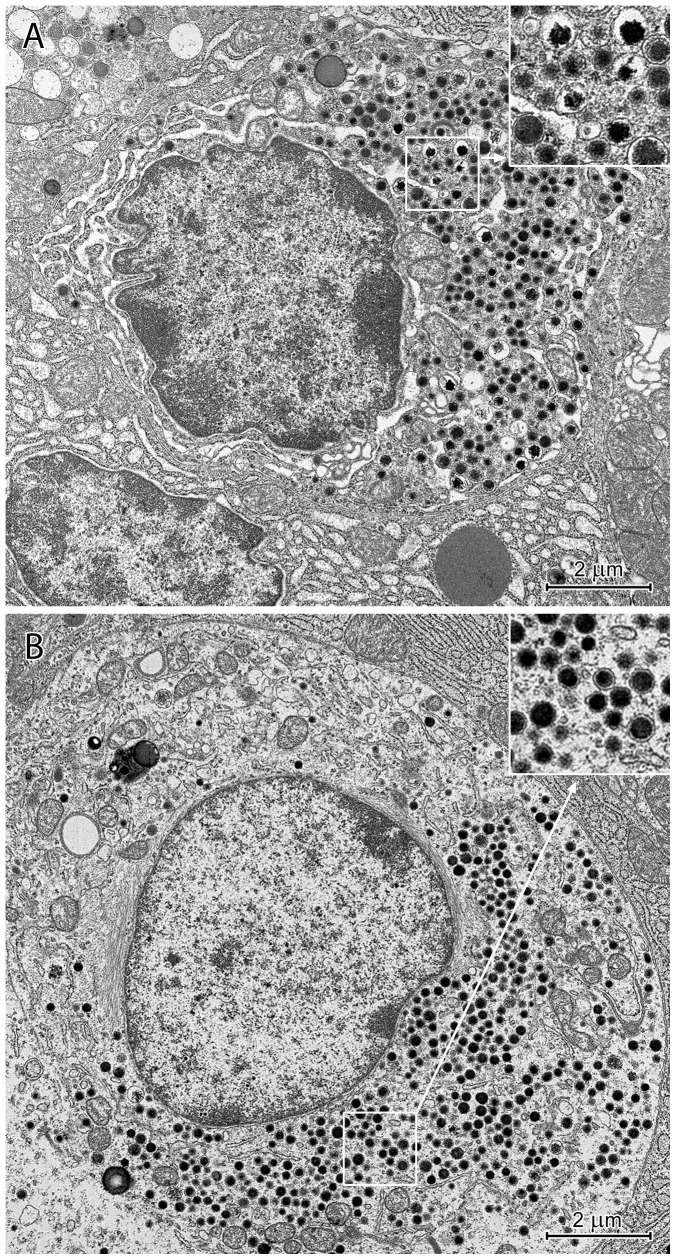
Ultrastructure of endocrine cells in the proper gastric gland of the corpus mucosa. (A) Endocrine cell with well-developed endoplasmic reticulum and polymorphic granules. (B) Endocrine cell with small, round, electron-dense granules and numerous filaments.

The base of the proper gastric glands contained chief cells (Figure 11A, B, C), parietal cells, and endocrine cells (Figure 12). Chief cells had a prominent bipolar organization, which resulted from numerous parallel sub- and paranuclear RER cisternae (Figure 11A, C). These cells also had an abundance of supranuclear secretory granules. Morphologically, secretory granules in the chief cells were heterogeneous with varying electron density and size; however, round, electron-dense granules were most common (Figure 11B). A prominent Golgi apparatus was visible in the supranuclear cytoplasm of the chief cells (Figure 11A). Parietal cells in the base of the gastric glands were similar to those in the neck.

Four types of endocrine cells could be distinguished using ultrastructural criteria. The most common were cells with a well-developed endoplasmic reticulum and small (130–300 nm) polymorphic granules (Figure 12A) as well as cells with numerous filaments and small (80–150 nm), round, electron-dense granules (Figure 12B). Cells with polymorphic, medium-sized (200–350 nm) granules and cells with homogeneous, medium-sized (200–350 nm), granules were also found.

#### Proper gastric glands in the CGG

**Figure 13:**
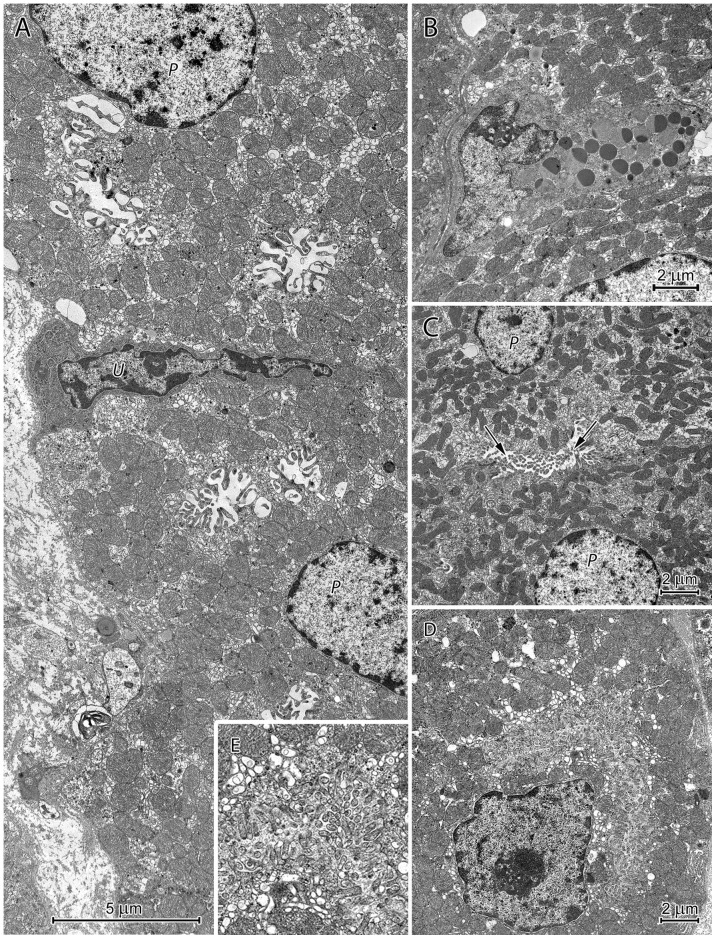
Ultrastructure of the neck of a proper gastric gland in the CGG. (A) Longitudinal section through the neck. Parietal cells - *P* situated above each other and undifferentiated cells - *U* between them. (B) Mucous neck cell. Note its small size and bipartite secretory granules. **C**. Two parietal cells - *P* forming the intercellular canaliculus (arrows). (D, E) Well-developed tubulovesicular system in the parietal cell at low (D) and high (E) magnification.

The upper regions of proper gastric glands in the CGG were primarily comprised of parietal and undifferentiated cells (Figure 13A). Mucous neck cells were sporadically found, and those observed were very small and contained almost exclusively bipartite secretory granules (Figure 13B). Due to their cubic shape, the parietal cells had large apical surfaces with prominent entrances into the tubulovesicular system. Neighboring parietal cells formed intercellular canaliculi connected to the gland's lumen (Figure 13C). Compared to the parietal cells in the corpus mucosa, those in the CGG had more prominent tubulovesicular system (Figure 13D, E). The undifferentiated cells were small, elongated, and had sparse cytoplasm with very few organelles, except for free ribosomes and RER (Figure 13A).

**Figure 14:**
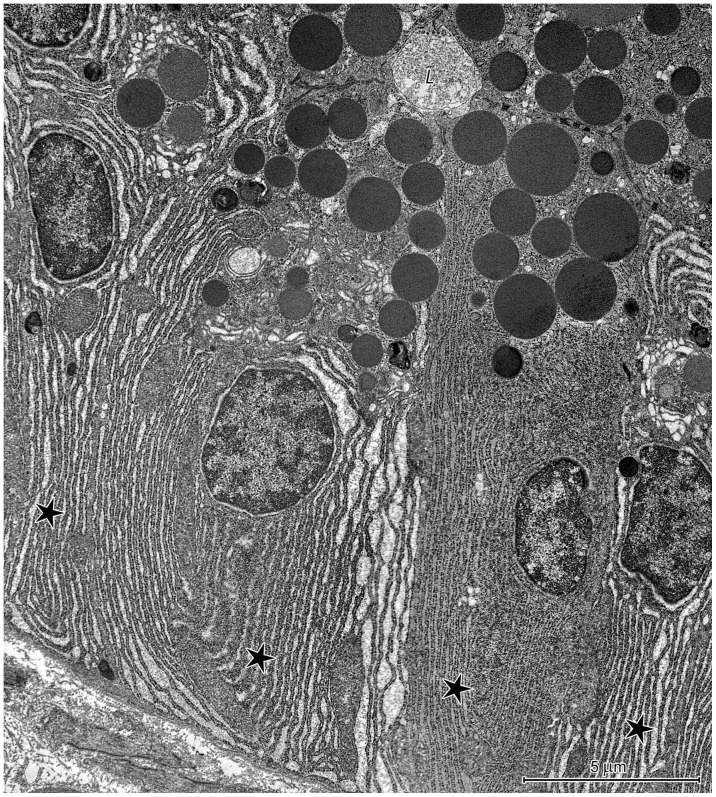
Ultrastructure of chief cells in the CGG. Note the well-developed rough endoplasmic reticulum (asterisks) with long cisternae that are parallel to the cell axis as well as numerous electron-dense secretory granules. L: the lumen of gland.

The basal regions of the CGG proper gastric glands consisted of chief cells, parietal cells, and endocrine cells. The majority of chief cells in the CGG could be distinguished from those in the mucosa by conspicuous RER and the almost exclusive presence of homogeneous, electron-dense secretory granules (Figure 14). Parietal cells were large, triangular, and had well-developed tubulovesicular system. The ultrastructure of endocrine cells in the CGG was similar to those in the corpus mucosa.

#### Pyloric glands

**Figure 15:**
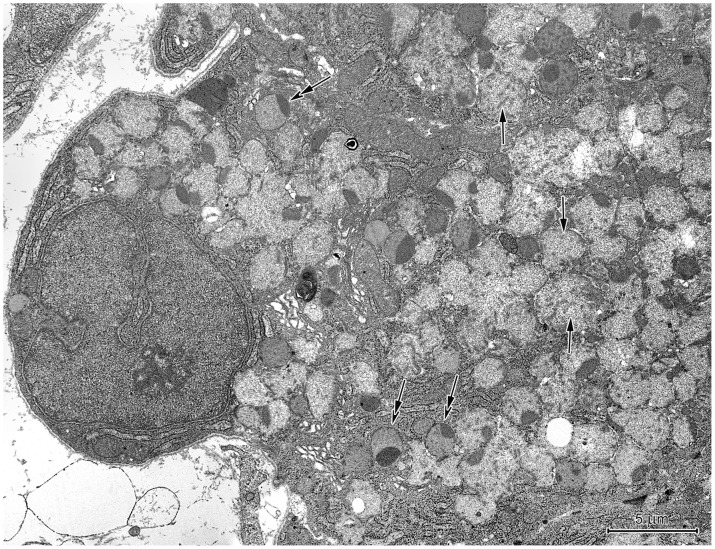
Ultrastructure of mucous cells in the pyloric glands. Note the three types of granules: 1) irregular, filled with electron-lucent material and “speckled” with electron-dense spots (arrows); 2) round, bipartite (double arrows); and 3) granules with intermediate properties.

The pyloric glands were comprised of mucous and endocrine cells. The mucous cells had basally situated, round or oval nuclei with deep nuclear envelope invaginations. Their cytoplasm contained three types of secretory granules: 1) irregularly shaped granules filled with electron-lucent material and “speckled” with electron-dense spots; 2) round, bipartite granules, similar to those in the mucous neck cells of the corpus; and 3) granules with intermediate properties between the other two types (Figure 15). “Speckled” granules were most common and sometimes formed larger aggregates. The mucous cells had a medium-sized, supranuclear Golgi apparatus, and their RER cisternae and mitochondria were scattered between the secretory granules. In addition to the endocrine cells described in the corpus mucosa, cells with large (400–450 nm), round, electron-dense granules were also present.

## Discussion

The European beaver stomach has several unique morphological features, including: 1) a CGG; 2) different cellular organization of proper gastric glands in the CGG and the mucosa; 3) striated muscles in the muscularis; and 4) specific mucus coating the luminal surface of the stomach.

The CGG was the most prominent gross morphologic feature of the European beaver stomach. To our knowledge, a CGG has been reported in twelve other mammalian species: the wombat [6], koala [7], dusky and northern shrew opossums [8], pangolins [9], [10], grasshopper mouse [11], dugong [12], and all species of manatees [13]. Animals possessing a CGG are herbivores with the exception of the grasshopper mouse and pangolin, which are insectivores. In the latter two species, the stomach mucosa is lined with stratified squamous epithelium. In contrast, the stomach mucosa of the other mammals mentioned above is lined with simple columnar epithelium.

The location and macroscopic structure of the CGG have significant interspecies differences [6], [7], [8], [9], [10], [11], [12], [13]. In the koala, wombat, dusky and northern shrew opossums, the CGG is present along the lesser curvature, directly behind the esophageal entrance. This is consistent with that in European and Canadian beavers. In contrast, in the grasshopper mouse, pangolins, and manatees, the CGG is located along the greater curvature and at a considerable distance from the esophageal entrance. In most species, the CGG is situated inside the stomach wall. However, in the grasshopper mouse and manatees, the CGG forms a prominent, elongated pouch on the greater curvature of the stomach [7], [11], [13].

In the wombat and koala, the secretory products of the CGG are released into the stomach lumen via 30 crater-like orifices. The dusky and northern shrew opossums have 40 to 60 CGG orifices [6], [7], [8], while the grasshopper mouse, pangolins, and manatees possess a single CGG orifice [7], [11], [13]. We found that the CGG of the European beaver drains into the stomach lumen via 12 main and 24-28 lateral apertures.

The general histological organization of the CGG is similar across all species [4], [6], [7], [8], [9], [10], [13]. The gland is formed by sac-like invaginations of the mucosa into the submucosa, which are usually branched. These invaginations are lined by surface epithelium and have numerous proper gastric glands. In the Malayan pangolin, the CGG also contains a few mucous glands [10].

The present study demonstrated important differences in the cellular composition between proper gastric glands located in the CGG and the mucosa of the European beaver stomach. The CGG glands were largely devoid of mucous neck cells, which were abundant in the mucosa glands. In addition, parietal cells were extremely abundant in the proper gastric glands in the CGG and formed intercellular canaliculi in the necks of the glands. The abundance of parietal cells in the CGG glands have also been described in the Canadian beaver [4]. Unfortunately, the presence of mucous neck cells was not previously analyzed in that species. Numerous parietal cells in CGG proper gastric glands have been observed in the wombat [7] and Florida manatee [13]. In contrast to our findings, a considerable number of mucous neck cells was reported in the CGG of both species. The abundance of parietal cells was also observed in the CGG of the koala; those cells were sandwiched with mucous cells in the upper regions of the CGG proper gastric glands [7].

In this study, we observed numerous well-developed chief cells in the basal regions of the CGG proper gastric glands in the European beaver. Quantitative analysis showed that the number of parietal and chief cells in the CGG are almost equal.

The nearly complete absence of mucous neck cells in the CGG proper gastric glands of the European beaver generates question about chief cell formation. It is generally accepted that chief cells arise from mucous neck cells via transdifferentiation [14], [15], [16], [17], [18]. This phenomenon includes reorganization of structure and biochemistry of cells during their migration from the neck to the base of gastric gland [19], [20]. As a consequence of this transformation, several transitional forms between mucous neck cells and mature chief cells have been described. These forms differ mainly in secretory granule morphology as well as the amount and organization of RER [17]. Transcription factors responsible for the specific architecture of chief cells have been recently identified. MIST1 has been shown to be necessary for proper structure of the apical parts in these cells and the formation of large secretory vesicles [19], and XBP1 has been shown to control organization of RER [20]. Chief cell formation is also strongly influenced by parietal cells [19]. Disturbances in chief cell development can result in mucosal metaplasia and cancer [21].

Our observations suggest that two different processes lead to chief cell formation in the European beaver stomach. The first one occurs in the CGG proper gastric glands and involves: 1) the proliferation of stem cells in the isthmus; 2) the migration of phenotypically undifferentiated cells through the neck; and 3) the chief cells formation from undifferentiated cells. The stage of well-developed, functionally active mucous neck cells is lacking in this process. The formation of chief cells from undifferentiated cells takes place at the border between the neck and base of the gland. The second process of chief cell formation in the European beaver stomach is that observed in other species [17]. This process includes the development of mucous neck cells and then transdifferentiation into chief cells. In the European beaver stomach, this transdifferentiation primarily occurs in proper gastric glands of the mucosa, resulting in the presence of numerous transitional cell forms. Thus, the beaver stomach could be an interesting model for studies of cellular differentiation in proper gastric glands since it includes both the classical and this proposed chief cell differentiation process.

Another unusual feature of the beaver stomach is the presence of striated muscle fibers in the muscularis covering the CGG. The stomach muscularis in most mammals is comprised exclusively of smooth muscle cells that extend in three directions: longitudinal, circular, and oblique. In the wombat stomach, striated fibers originate at the esophagus, run parallel to the longitudinal axis of the organ, and terminate near the pyloric border of the CGG [6].

In the stomach of the American beaver, a triangular muscular septum, which divides the gastric cavity into two parts, has been reported [1]. Similarly, a prominent muscular ridge, which separates the stomach lumen into superior and inferior regions, has also been described in the Florida manatee [13], [22]. In this species, the ridge is formed by oblique fibers of the tunica muscularis and likely ensures that the stomach's entire content is exposed to CGG secretions. We did not observe an equivalent structure in the European beaver stomach here.

We found that a very thick layer of specific mucus covered the stomach mucosa in the European beaver. Mucus thickness was measured using frozen sections of the stomach wall in the corpus region and ranged from 400 to 950 µm. For comparison, the mucus thicknesses of unfixed, cryostat sections of the stomach fundus in rats, rabbits, and pigs has been reported to be 31.3±11.4, 155.1±85.5, and 190.7±80.7 µm, respectively [23]. Lower mucus thicknesses (ca. 50–70 µm) were found by Varum et al. [24] in studies of frozen pig stomachs sections.

It should be emphasized that conventional histological techniques, employing organic solvents and paraffin, usually result in mucus dehydration and shrinkage [25], [26], [27]. Despite the use of these aggressive procedures in our study, the mucus layer with a thickness up to 400 µm was still observed. This suggests that the mucus in the beaver stomach is highly chemical resistant. In addition, the arrangement of cellular debris in regular rows extending from areas of epithelial cell exfoliation to the stomach lumen was not disturbed by mechanical digestive forces, indicating a semisolid mucus consistency.

Electron microscopy showed changes in gastric pit cells during their life cycle, which allowed us to form a preliminary hypothesis about mucus formation in the beaver stomach. Five life-cycle stages could be identified in the pit cells in the corpus region. The first stage was observed in the youngest pit cells that lie in the lowest regions of the gastric pits. These cells contain small to moderate numbers of round, electron-dense granules in their apical regions. The contents of these granules are released by exocytosis. Beaver pit cells in this phase of development are similar to those of other species [28], [29]. In the second life-cycle stage, electron-dense granules are more numerous and form a large, supranuclear accumulation. A narrow rim of cytoplasm separates this accumulation from the apical cell membrane. The third life-cycle stage is characterized by changes in granule morphology (increased size, changes in shape, and decreased electron density) and granule aggregation. The mechanisms driving this phenomenon appear to be similar to those responsible for the transformation of mucus granules in goblet cells [30]. In the fourth life-cycle stage, cellular organelles degenerate, and the apical cell membrane loses continuity, partially releasing the mucus mass from the cell. The volume of mucus accumulated inside the cells appears markedly increased, probably due to hydration. The last life-cycle stage occurs in the upper regions of the gastric pits and includes the exfoliation of degenerated cells and their incorporation into the mucus layer.

We noted slight differences in the ultrastructure of pit cells situated in the corpus region and those in the pyloric region. The cells in the bottom and middle regions of the pyloric gastric pits contained numerous granules with variable electron density; the contents of these granules were released by exocytosis. In the upper regions of the gastric pits, cells underwent changes similar to those observed in the corpus region. The presence of numerous cells releasing their granule contents via exocytosis is probably related to the length of the pits in the pyloric region. These pits are very deep and occupy about 80% of the mucosa thickness.

Our observations suggest that mucus in the beaver stomach is formed by two secretory processes: merocrine, where granule content is released by exocytosis and holocrine-like, where granules accumulate in the cytoplasm and form a mucus mass that is incorporated with destroyed cells into the mucus coat. We failed to find any other mammalian species with a mucus stomach coat similar to that of the beaver, which is created by complex gastric pit cell transformations. However, in our opinion, some similarities exist between formation of mucus coat in the beaver stomach and “honeycomb” in the babirusa stomach [31]. The “honeycomb”, overlying the gastric pits in the cardiac region, consists of hexagonal tubes build by connected each other, exfoliated pits cells and is colonized by numerous bacteria [31].

There are two possible explanations for the functional significance of mucus in the European beaver stomach. First, the thick layer of specific mucus likely acts as a barrier against physical injuries to the mucosa, such as those caused by the beaver's diet. The mucus also protects the epithelium against hydrogen ions and pepsin. Second, the thick mucus layer, especially in the pyloric region, may form a niche for microorganisms and provide optimal conditions (i.e., more neutral pH) for their development [25], [32]. The most frequently discussed function of stomach mucus microorganisms is bacterial fermentation. The bacteria ferment the structural polysaccharides of plants using enzymes that the host cannot produce [32], [33]. This aspect of beaver stomach physiology is completely unknown and requires additional studies.

At least four types of endocrine cells were found in the proper gastric glands of both the CGG and corpus mucosa in the European beaver. To date, the ultrastructure of CGG endocrine cells has only been investigated in the koala [34], where three cell types were identified. Immunohistochemical studies of endocrine cell distribution in the CGG have been conducted in the koala and Malayan pangolin [35], [36]. Those studies found cells that were immunoreactive to somatostatin, pancreatic polypeptide, serotonin, and glucagon in both animals. However, the number and localization of these cells differed between species.

The pyloric glands in the beaver stomach contained endocrine cells similar to those in proper gastric glands as well as cells with large, round, electron-dense granules. These cells likely correspond to the cells with an acidophilic cytoplasm, which were visible in H&E-stained sections. The large cells with basophilic cytoplasm observed in histological pyloric sections were not identified using electron microscopy.

Our results provide the foundation for future physiological studies of beaver digestion processes. We identified many unique morphological features in the European beaver stomach. However, our findings also had more general scientific significance. We showed that the beaver stomach can be an interesting model for studies of chief cell differentiation and the biology of mucus producing cells.
